# Bilateral Renal Infarct in a Young Adult: Unveiling an Autoimmune Enigma

**DOI:** 10.7759/cureus.59095

**Published:** 2024-04-26

**Authors:** Sheima Gaffar, Dhayanithi Dhayalan, Han Li, Mohankumar Doraiswamy, Naveen Baskaran

**Affiliations:** 1 Internal Medicine, Sree Mookambika Institute of Medical Sciences, Kulasekharam, IND; 2 Nephrology, Kaweah Health Medical Center, Visalia, USA; 3 College of Medicine, University of Florida, Gainesville, USA; 4 Internal Medicine, Mercy Hospital Northwest Arkansas, Rogers, USA; 5 Internal Medicine, Monmouth Medical Center, Long Branch, USA; 6 Internal Medicine, University of Florida, Gainesville, USA

**Keywords:** blood hypercoagulability, congenital cardiac anomalies, left ventricular non-compaction cardiomyopathy (lvnc), left ventricular non-compaction cardiomyopathy, renal cortical infarct, renal infarct

## Abstract

A man in his late 20s presented to the emergency department with sudden-onset abdominal pain. Urinalysis was significant for hematuria and slightly elevated creatinine. A computed tomography (CT) scan with IV contrast revealed bilateral renal infarcts, which was corroborated by a computed tomography angiogram (CTA). Further evaluation by an autoimmune panel demonstrated a positive antinuclear antibody, while echocardiography showed left ventricular non-compaction cardiomyopathy. The workup included consultations with multiple specialities and additional investigations to assess hypercoagulability, vasculitis, and infectious etiologies. Following supportive care, the patient was discharged in stable condition with a plan for outpatient follow-up and further workup, including screening of first-degree family members for left ventricular non-compaction and associated cardiovascular risks. Here we describe a report of a rare case of bilateral renal infarct of possible thromboembolic etiology due to an underlying rare genetic cardiovascular condition.

## Introduction

Bilateral renal infarct is a rare condition characterized by the occlusion of the renal arteries or their branches, leading to ischemic damage in both kidneys. Patients with renal infarction typically present with abdominal or flank pain, nausea, vomiting, and hematuria. Performing a contrast-enhanced study can expedite the correct diagnosis and facilitate prompt initiation of therapy and evaluation for underlying causes. The use of advanced imaging techniques has increased the detection of incidental cases with non-specific symptoms.

Common causes include emboli from the heart, in-situ thrombosis, and interventional procedures. Other contributing factors include coagulation disorders, vasculitis, connective tissue diseases, endocarditis, atherosclerosis, aortic aneurysms, smoking, alcohol, and trauma. A relatively rare cause of renal infarction is embolism from left ventricular non-compaction cardiomyopathy (LVNC). We report a case of bilateral embolic renal infarcts in a patient with LVNC.

## Case presentation

A man in his late 20s presented to the emergency department with one day of acute onset severe abdominal pain with radiation to bilateral flanks, nausea, and one episode of vomiting. The pain was a stabbing sensation, with no variation in position or oral intake. He denied urinary symptoms, fever, chills, shortness of breath, or dyspnea on exertion. There was no significant past medical history. The patient drank 12 shots and smoked four cigars daily. Their family history was notable for the sudden cardiac death of his maternal uncle in the late 20s. On admission, his vital signs were significant for tachycardia (102/min), tachypnea (28/min), elevated BP (159/114 mmHg), and a pulse oximetry of 94% on room air.

A urine sample was obtained, and urinalysis showed small hematuria and 30 mg/dL of protein. Blood investigations revealed elevated CRP levels of 24.7 mg/dL (normal: <10 mg/dL) and leukocytosis. Renal function tests showed a blood urea nitrogen of 32, creatinine of 1.33, and an estimated glomerular filtration rate (eGFR) of 66. Liver function tests were normal. A venous blood gas indicated a pH of 7.41 and HCO3 of 25. Lactate dehydrogenase (LDH) was elevated (758 IU/L, normal: 140-280 IU/L). An electrocardiogram showed sinus tachycardia with LV hypertrophy and QT prolongation.

**Figure 1:**
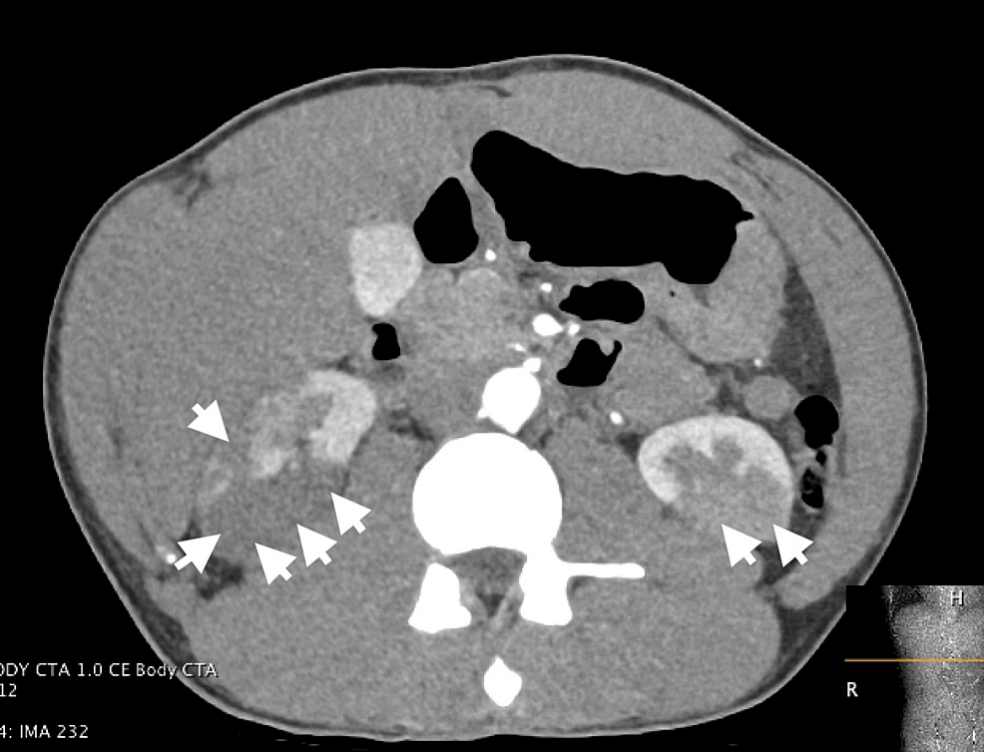
CT abdomen showing bilateral renal infarcts and high-grade stenosis of the inferior segmental branch of the right renal artery and narrowing of inferior segmental branches.

The right upper quadrant ultrasound showed no significant abnormalities. However, a CT abdomen with IV contrast revealed bilateral renal infarcts, with the right side more affected than the left (Figure 1). Additionally, there was high-grade stenosis of the inferior segmental branch of the right renal artery and narrowing of inferior segmental branches, raising concerns for embolism or vasculitis. A vasculitis workup including antineutrophil cytoplasmic antibodies (ANCA) (both myeloperoxidase (MPO) and proteinase 3 (PR3)) and double-stranded DNA (dsDNA) was negative. Magnetic resonance angiography (MRA) of the abdomen and pelvis did not show evidence of a vasculitic cause of the infarcts, such as polyarteritis nodosa. A hypercoagulability workup was completely negative.

A portable chest X-ray showed cardiomegaly. Transthoracic echocardiography revealed severe LV systolic dysfunction (ejection fraction (EF) 10-15%), LV segment hypokinesia, and moderate right ventricular systolic dysfunction. Cardiac MRI showed, in addition to the above, prominent LV apical trabeculations with a noncompaction-to-compaction ratio of 3, consistent with LVNC. Right heart catheterization (RHC) showed significantly elevated filling pressures, while left heart catheterization (LHC) was unremarkable with normal coronaries.

Blood cultures, tracheal culture, acid-fast bacteria (AFB) staining, and urine culture were negative. Immunology assays showed a significantly positive antinuclear antibody (ANA) titer of 1:320 with a speckled pattern and elevated ribonucleic protein IgG levels (62 units, normal <20). Additional autoimmune workup, including anti-Jo1/KU/MI-2/MPO/PL-12/RF/PR3/Scl70/NXP/Smith/MDA-5, was negative.

In the absence of infection, vasculitis, and hypercoagulopathy and with the imaging findings of LVNC and family history of sudden cardiac death, the leading diagnosis was bilateral renal infarcts from LVNC. The patient had imaging consistent with renal infarct and LVNC. Due to intracardiac turbulent flow, LVNC predisposes to a greater risk of embolism and sudden cardiac death [1]. However, another possible diagnosis was an autoimmune process. Specifically, systemic lupus erythematosus (SLE) or mixed connective tissue disease (MCTD) could align with the renal involvement and were suggested by the positive ANA and anti-ribonucleoprotein (anti-RNP) antibodies. However, the patient lacked the characteristic musculocutaneous, hematologic, or neurologic findings, and subsequent autoimmune workup, including anti-dsDNA, was negative. Ultimately, the association of LVNC with embolism and the inability to meet the full diagnostic criteria for autoimmune disease led us to favor LVNC as the underlying diagnosis for his bilateral renal infarcts.

Coupled with supportive measures, the patient was initially treated with a heparin drip given bilateral renal infarcts. Given the high initial suspicion of vasculitis, treatment was initiated with IV methylprednisolone 1g daily for three days. This transitioned to oral prednisone 80 mg once daily with a gradual taper. With these treatments, his symptoms gradually improved.

Following discharge, a subsequent angiogram of the celiac, superior mesenteric, and bilateral renal arteries did not show vasculopathy. Although his kidney function recovered to baseline, his heart failure progressed over the following year with worsening fatigue, dyspnea, and diuresis requirements; his EF was 15-20% one year later.

## Discussion

Renal infarction is an arterial vascular event that may cause irreversible damage to kidney tissues. It is a very rare condition in adults, with an estimated incidence of nearly 0.007% to 0.05% in the general population [2]. A review of autopsy records revealed an incidence of renal infarcts in 1.4% of cases [3]. It should be considered in patients with acute abdominal pain, particularly when associated with risk factors such as hypercoagulable states, atrial fibrillation, or embolic sources [4]. The estimated incidence of renal infarction was 0.004% in patients presenting with acute abdominal pain to ED; of which 50% were correctly diagnosed in the initial ED visit [5]. No specific clinical characteristics could be identified to distinguish those patients diagnosed early and those with delayed diagnosis [6]. Clinical characteristics commonly include abdominal and/or flank pain, elevated LDH, and elevated CRP [4]. Imaging techniques such as contrast-enhanced CT play a crucial role in confirming the diagnosis [7].

Our case was a unique presentation of bilateral renal artery infarcts secondary to LVNC, a relatively rare congenital disease defined by a two-layered myocardium comprising a non-compacted layer with prominent trabeculations and a thin compacted layer. LVNC is a genetically heterogeneous disease, associated with multiple pathogenic variants in sarcomere function [8]. LVNC is associated with cardiac failure, stroke, mesenteric artery occlusion, lethal arrhythmias, and mural thrombi [9-11]. Heart failure is the most common presenting symptom (68%), and arrhythmias are observed in 41% of patients. One case described renal infarct in LVNC but was in a middle-aged female with unilateral renal infarct [12]. This patient was managed with anticoagulation and pulse steroid therapy with dramatic improvement. Diagnosis can be established by echocardiography based on multiple criteria emphasizing intratrabecular recess depth, non-compaction-to-compaction ratio, or the number of trabeculations [13]. For example, the Jenni et al. criteria consist of (1) a two-layered myocardium with a non-compacted-to-compacted ratio >2; (2) noncompaction predominantly in the mid-lateral, apical, and mid-inferior regions; (3) intertrabecular perfusion on color Doppler; and (4) absence of coexisting abnormalities [13]. In equivocal cases, cardiac MRI can support the diagnosis. Although the echocardiograph interpretation did not initially consider noncompaction cardiomyopathy in our case, it ruled out coexisting abnormalities and demonstrated heart failure in our patient, with LNVC subsequently diagnosed on cardiac MRI. It is posited that at least eight subtypes of LVNC exist, including dilated LVNC, hypertrophic LVNC, hypertrophic dilated LVNC, biventricular LVNC, LVNC with arrhythmia, benign LVNC, restrictive LVNC, and LVNC with congenital heart disease [14]. However, as a rare diagnosis, LVNC remains largely uncharacterized in terms of epidemiology, prognosis, and complications. This case further characterizes the clinical presentation of LVNC and emphasizes the role of family history and prompt imaging in diagnosing LVNC-causing renal infarcts.

## Conclusions

Though bilateral renal infarct is rare, its presentation in young adults with multi-system findings and without significant past medical history is both difficult to diagnose and potentially debilitating, demanding a multi-disciplinary approach. LVNC is a rare cause of renal thromboembolism and infarct that was diagnosed in our patient by cardiac MRI and echocardiography. Young patients with findings suggesting of LVNC may require a cardiac MRI for diagnosis. Prompt diagnosis of LVNC and its complications in young patients with unprovoked embolism and family history of sudden death may facilitate treatment and further disease characterization.
